# CSGID Solves Structures and Identifies Phenotypes for Five Enzymes in *Toxoplasma gondii*

**DOI:** 10.3389/fcimb.2018.00352

**Published:** 2018-10-05

**Authors:** Joseph D. Lykins, Ekaterina V. Filippova, Andrei S. Halavaty, George Minasov, Ying Zhou, Ievgeniia Dubrovska, Kristin J. Flores, Ludmilla A. Shuvalova, Jiapeng Ruan, Kamal El Bissati, Sarah Dovgin, Craig W. Roberts, Stuart Woods, Jon D. Moulton, Hong Moulton, Martin J. McPhillie, Stephen P. Muench, Colin W. G. Fishwick, Elisabetta Sabini, Dhanasekaran Shanmugam, David S. Roos, Rima McLeod, Wayne F. Anderson, Huân M. Ngô

**Affiliations:** ^1^Pritzker School of Medicine, University of Chicago, Chicago, IL, United States; ^2^Center for Structural Genomics of Infectious Diseases and the Department of Biochemistry and Molecular Genetics, Feinberg School of Medicine, Northwestern University, Chicago, IL, United States; ^3^Department of Ophthalmology and Visual Sciences, University of Chicago, Chicago, IL, United States; ^4^Illinois Math and Science Academy, Aurora, IL, United States; ^5^Strathclyde Institute of Pharmacy and Biomedical Sciences, University of Strathclyde, Glasgow, United Kingdom; ^6^Gene Tools, LLC, Philomath, OR, United States; ^7^Department of Biomedical Sciences, College of Veterinary Medicine, Oregon State University, Corvallis, OR, United States; ^8^Department of Molecular Biology and Biotechnology, University of Sheffield, Sheffield, United Kingdom; ^9^School of Biomedical Sciences, Faculty of Biological Sciences, and Astbury Centre for Structural Molecular Biology, University of Leeds, Leeds, United Kingdom; ^10^School of Chemistry and Astbury Centre for Structural Molecular Biology, University of Leeds, Leeds, United Kingdom; ^11^Biochemical Sciences Division, CSIR National Chemical Laboratory, Pune, India; ^12^Department of Biology, University of Pennsylvania, Philadelphia, PA, United States; ^13^Department of Pediatrics (Infectious Diseases), Institute of Genomics, Genetics, and Systems Biology, Global Health Center, Toxoplasmosis Center, CHeSS, The College, University of Chicago, Chicago, IL, United States; ^14^BrainMicro LLC, New Haven, CT, United States

**Keywords:** *Toxoplasma gondii*, PPMO, phosphoglycerate mutase, nucleoside diphoshate kinase, ribulose-3-phosphate epimerase, ribose-5-phosphate isomerase, ornithine aminotransferase, crystallography

## Abstract

*Toxoplasma gondii*, an Apicomplexan parasite, causes significant morbidity and mortality, including severe disease in immunocompromised hosts and devastating congenital disease, with no effective treatment for the bradyzoite stage. To address this, we used the Tropical Disease Research database, crystallography, molecular modeling, and antisense to identify and characterize a range of potential therapeutic targets for toxoplasmosis. Phosphoglycerate mutase II (PGMII), nucleoside diphosphate kinase (NDK), ribulose phosphate 3-epimerase (RPE), ribose-5-phosphate isomerase (RPI), and ornithine aminotransferase (OAT) were structurally characterized. Crystallography revealed insights into the overall structure, protein oligomeric states and molecular details of active sites important for ligand recognition. Literature and molecular modeling suggested potential inhibitors and druggability. The targets were further studied with vivoPMO to interrupt enzyme synthesis, identifying the targets as potentially important to parasitic replication and, therefore, of therapeutic interest. Targeted vivoPMO resulted in statistically significant perturbation of parasite replication without concomitant host cell toxicity, consistent with a previous CRISPR/Cas9 screen showing PGM, RPE, and RPI contribute to parasite fitness. PGM, RPE, and RPI have the greatest promise for affecting replication in tachyzoites. These targets are shared between other medically important parasites and may have wider therapeutic potential.

## Introduction

*Toxoplasma gondii* is one of the most significant parasites that impacts human health, with estimates that as many as one third to one half of the human population are infected (Montoya and Liesenfeld, 2004; Furtado et al., 2011; Torgerson and Mastroiacovo, 2013; Flegr et al., 2014; McLeod et al., 2014; Lykins et al., 2016). A relative of the parasite that causes malaria, *T. gondii* is an intracellular parasite that has two major life stages in humans, tachyzoites and bradyzoites. Tachyzoites cause acute infection, while bradyzoites are the encysted, dormant life stage responsible for reactivation disease. While treatment is available for the acute infection, there is currently no effective medication for the bradyzoite stage (McLeod et al., 2014). Additionally, parasites can be passed to a fetus *in utero* when a pregnant woman is acutely infected during gestation. This can cause chorioretinitis and neurological complications in the fetus (McLeod et al., 2012). Moreover, there is increasing understanding of the potential long-term sequelae of chronic infection with *T. gondii* on risk of neurodegenerative disease and malignancy (Ngô et al., 2017). Treatment for active infection exists but is limited by toxicity and hypersensitivity. Thus, new therapeutic targets and medicines are needed, with several potential solutions in development (Zhou et al., 2014; McPhillie et al., 2016; Sidik et al., 2016).

At the Center for Structural Genomics of Infectious Diseases (CSGID), the first *Toxoplasma* Structural Genomics Pipeline was established. Subsequently, CSGID began selecting parasite proteins for structural characterization using established approaches capable of successful identification of potential drug targets, coupled with the Tropical Diseases Research (TDR) Database (Anderson, 2009; Crowther et al., 2010; Magariños et al., 2012). Herein, 5 soluble enzymes were selected for further study. This process was made possible due to the integration of large amounts of genomic, biochemical, and pharmacological data by the TDR Database, which provides evidence collectively generated by the scientific community concerning potential molecular targets and inhibitory compounds that have properties consistent with Lipinski's rules for orally available drugs (Lipinski, 2004). The targets studied herein were crystallized and their structures characterized, as structural studies have potential to inform molecular targeting and medicinal chemistry can facilitate development of novel anti-parasitic compounds.

We further hypothesized that using phosphorodiamidate morpholino oligomers linked to a cellular delivery moiety, such as either an octaguanidinium dendrimer [Vivo-Morpholinos (vivoPMOs)], or arginine-rich peptide, we would decrease expression of these enzymes, identified as potential drug targets by the *Toxoplasma* Structural Genomic Pipeline, in YFP-expressing *T. gondii* tachyzoites, and that down-regulation of these enzymes would result in decreased replication as quantified by fluorescent intensity. The approach of using morpholinos to target specific parasitic enzymes has been successful in previous studies (Lai et al., 2012; McPhillie et al., 2016). VivoPMOs are typically used to decrease gene expression by one of two different mechanisms, namely mechanical disruption of interactions between RNA and snRNP, thereby preventing splicing of introns, resulting in nonsense-mediated decay of the transcript and/or defective protein upon translation, and through direct prevention of translation by blocking interactions between mature mRNA and the ribosome. In preventing effective protein expression, we could determine whether a particular enzyme contributed to parasite replication, suggesting its potential as a therapeutic target.

Molecular transporters can deliver PMOs and small inhibitory molecules of therapeutic value. Transductive peptides or octaguanidinium dendrimer of a Vivo-Morpholino (Gene Tools, Philomath, Oregon) deliver PMOs or other molecules across cell membranes. Octaarginine can carry small molecules into the retina (McLeod et al., 2013). Similar arginine-rich cell-penetrating peptides can access other places where medication transport is problematic; for example, rabies virus glycoprotein-tagged small molecules are capable of passing through the blood-brain barrier and octaarginine-conjugated small molecules, for example, cross into encysted bradyzoites (Samuel et al., 2003; Liu et al., 2009).

The enzymes selected from the TDR database as small and tractable for expression and crystallization included: phosphoglycerate mutase II (hereafter referred to as PGM), nucleotide diphosphate kinase (NDK), ribulose phosphate 3-epimerase (RPE), ribose-5-phosphate isomerase (RPI), and ornithine aminotransferase (OAT). Information about candidate inhibitors of these apicomplexan enzymes is summarized in Table 1.

**Table 1:** Target enzyme characterization and candidate inhibitors.

**Target enzyme**	**Function**	**Pathogens in which it has been studied**	**Phenotypes/ Means of assay**	**Candidate inhibitors**	**References**
Phosphoglycerate Mutase	Catalyzes transition from 3-phosphoglycerate to 2-phosphoglycerate; important for glycolysis	*Trypanosoma* spp. *Toxoplasma gondii*	replication	Inositol hexakisphosphate, benzene hexacarboxylate, 2-hydroxy-4-phosphonobutanoate, epigallocatechin-3-gallate Xanthone derivatives	McAleese et al., 1985; Rigden et al., 1999; Chevalier et al., 2000; Opperdoes and Michels, 2001; Djikeng et al., 2007; Singh et al., 2013; Li et al., 2017; Wang et al., 2018
Nucleoside Diphosphate Kinase	Catalyzes movement of phosphate from nucleoside triphosphate to nucleoside diphosphate (GTP + ADP –> GDP + ATP)	*Plasmodium falciparum Leishmania amazonensis*	Stress response in RPS-13; replication	Adenosine-3-phosphate-5-phosphosulfate SU11652	Reyes et al., 1982; Schneider et al., 1998; Kolli et al., 2008; Motomura et al., 2014; Vieira et al., 2015
Ribulose Phosphate 3-Epimerase	Converts ribulose-5-phosphate into xylulose-5-phosphate (part of Calvin cycle in plants)	*Trypanosoma cruzi Plasmodium falciparum*	Involved in pentose-phosphate pathway and the generation of nucleotides; replication; in same pathway as the R5PI; implications for plastid	D-2-Deoxyribose 5-phosphate	Wood, 1979; Caruthers et al., 2005; Igoillo-Esteve et al., 2007
Ribose-5-Phosphate Isomerase	Catalyzes transition from ribose-5-phosphate to ribulose-5-phosphate (upstream of above enzyme)	*Trypanosoma cruzi*	important in growth phase; cell invasion; implications for plastid; cell death/ replication potentially (observed in Arabidopsis)	4-phosphoerythronate	Igoillo-Esteve et al., 2007
Ornithine Aminotransferase	Forms first intermediate in pathway to proline from ornithine (is reversible); been implicated in eye disease	*Plasmodium falciparum*	Replication	L-canaline, 5-fluoromethylornithine	Kito et al., 1978; Storici et al., 1999; Müller et al., 2009; Sturm et al., 2009; Kronenberger et al., 2014

## Methods

### Cloning, expression, and purification

**Figure 1:**
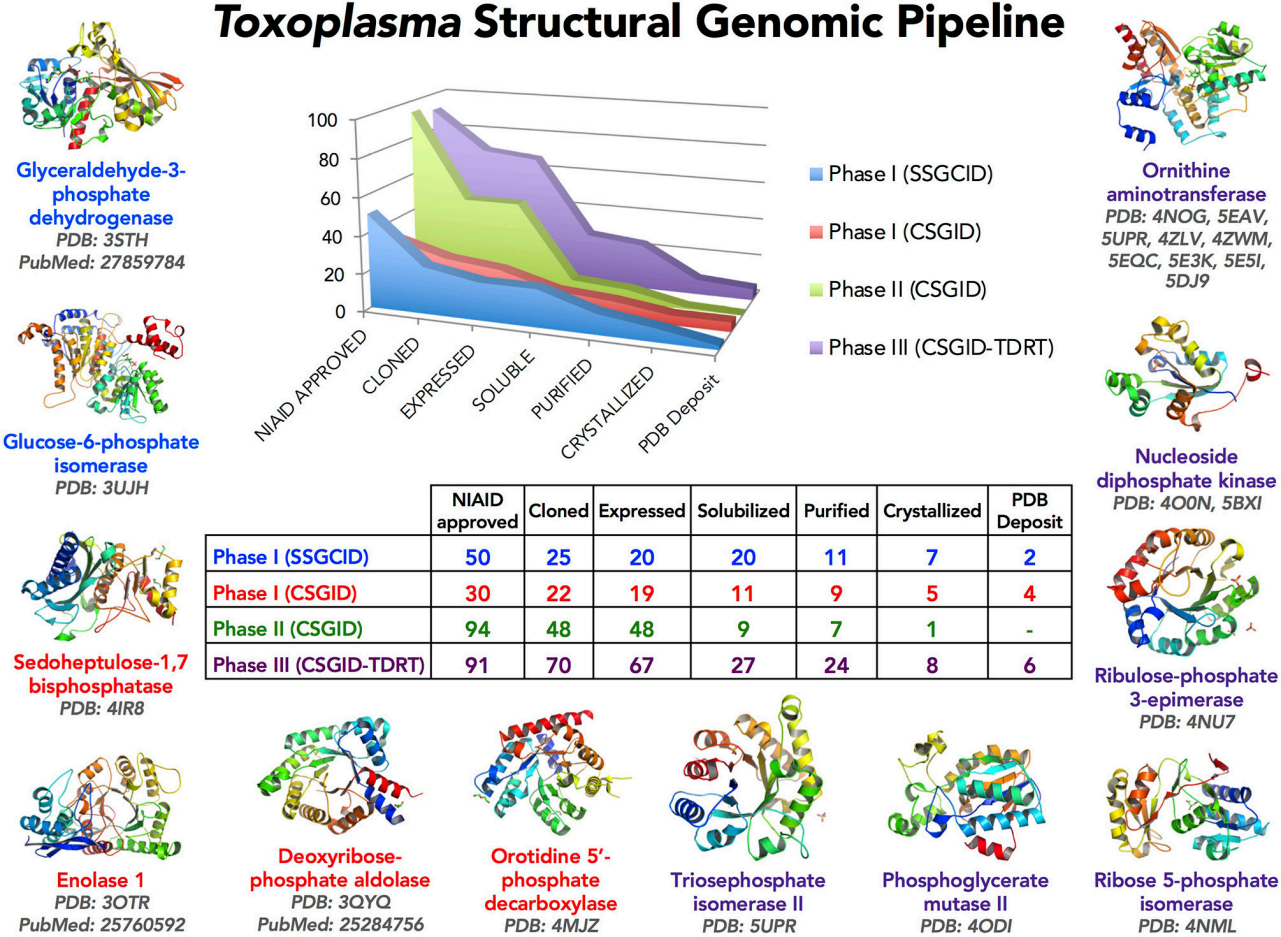
The *Toxoplasma* Structural Genomics Pipeline identified promising targets with good predicted druggability (Phase III) and outstanding interest to the *Toxoplasma* research community (Phase I, II). *Toxoplasma* proteins were selected for Phase I from published work up to 2009 and communicated with the research laboratories. Phase II included proteins from published and unpublished studies suggested by international researchers at the end of 2011. Phase III candidates were selected from the Tropical Diseases Research Database (TDRT). Proteins judged very unlikely to crystallize based on analysis by XtalPred were eliminated (Slabinski et al., 2007). Several structures solved have already been described (Ruan et al., 2015; Tonkin et al., 2015; Dubey et al., 2017). This crystallography pipeline remains available for production and solution of structures of proteins for scientists in the *Toxoplasma* research community.

Five genes from *T. gondii* ME49 (GI: 237843677, 237844373, 237835673, 237834547, and 237832613) corresponding to a putative phosphoglycerate mutase II (*Tg*PGM; residues 1–265 of the original CSGID entry; this entry was modified in 2012; see original NCBI Reference Sequence: XP_002371136.1), a putative nucleoside diphosphate kinase (*Tg*NDK) (residues 1–155), ribulose phosphate 3-epimerase (*Tg*RPE) (residues 1–230), a putative ribose 5-phosphate isomerase (*Tg*RPI) (residues 1–259), and a putative ornithine aminotransferase (*Tg*OAT) (residues 17–441), respectively, were cloned into the ligation-independent-cloning (Aslanidis and de Jong, 1990) isopropyl β-D-1-thiogalactopyranoside (IPTG)-inducible pMCSG28 vector for recombinant bacterial expression in BL21(DE3)/pMagic *Escherichia coli* (*E. coli*). All clones were obtained from J. Craig Venter Institute, a CSGID partner, and submitted into the CSGID protein production pipeline (Figure 1) as IDP92076 (*Tg*PGM), IDP92074 (*Tg*NDK), IDP92047 (*Tg*RPE), IDP92040 (*Tg*RPI), and IDP92102 (*Tg*OAT). The pMCSG28 vector possesses a purification tag including 6 × His affinity sequence and a Tobacco Etch Virus (TEV) protease cleavage site. The *E. coli* cells were induced with 1 mM IPTG at 25°C after the optical density of cells in culture flasks reached 0.6 at 600 nm under 37°C and constant aeration at 200 rpm. Terrific Broth (TB) (PGM, NDK, and RPE) and the Se-Met MCSG-M9 (Medicilon Inc.) (RPI) medium was used. Overnight induction was completed by collecting cells at 6,000 rpm, 4°C for 10 min. Cells' paste was resuspended in chilled Lysis Buffer [43 mM Na_2_HPO_4_, 3.25 mM citric acid, 250 mM NaCl, 100 mM ammonium sulfate, 5% glycerol, 5 mM imidazole, 1.5 mM magnesium acetate, 1 mM CaCl_2_, 0.08% n-Dodecyl β-D-maltoside (DDM), 5 mM β-mercaptoethanol (BME)] pH 7.8 followed by sonication on ice. Crude sonication mixture was centrifuged at 19,000 rpm, 4°C for 40 min to obtain soluble fraction containing target protein, which was applied onto a 5-ml Ni-NTA column (GE Healthcare, Piscataway, NJ) for purification. The column was washed with buffer containing 10 mM Tris-HCl pH 8.3, 500 mM NaCl, 25 mM imidazole and 5 mM BME to remove non-specifically bound *E. coli* proteins, followed by elution of target protein with 500 mM imidazole in the 10 mM Tris-HCl buffer pH 8.3 containing 500 mM NaCl and 5 mM BME (buffer A). A HiLoad™ 26/60 Superdex™ 200 column (GE Healthcare, Piscataway, NJ) was used to further purify target protein in the buffer A. Purity of all proteins was analyzed by SDS-PAGE. Pooled fractions were concentrated and stored at −80°C for further use or used in screening crystallization conditions.

### Crystallization, X-ray data collection, and structure determination

**Figure 2:**
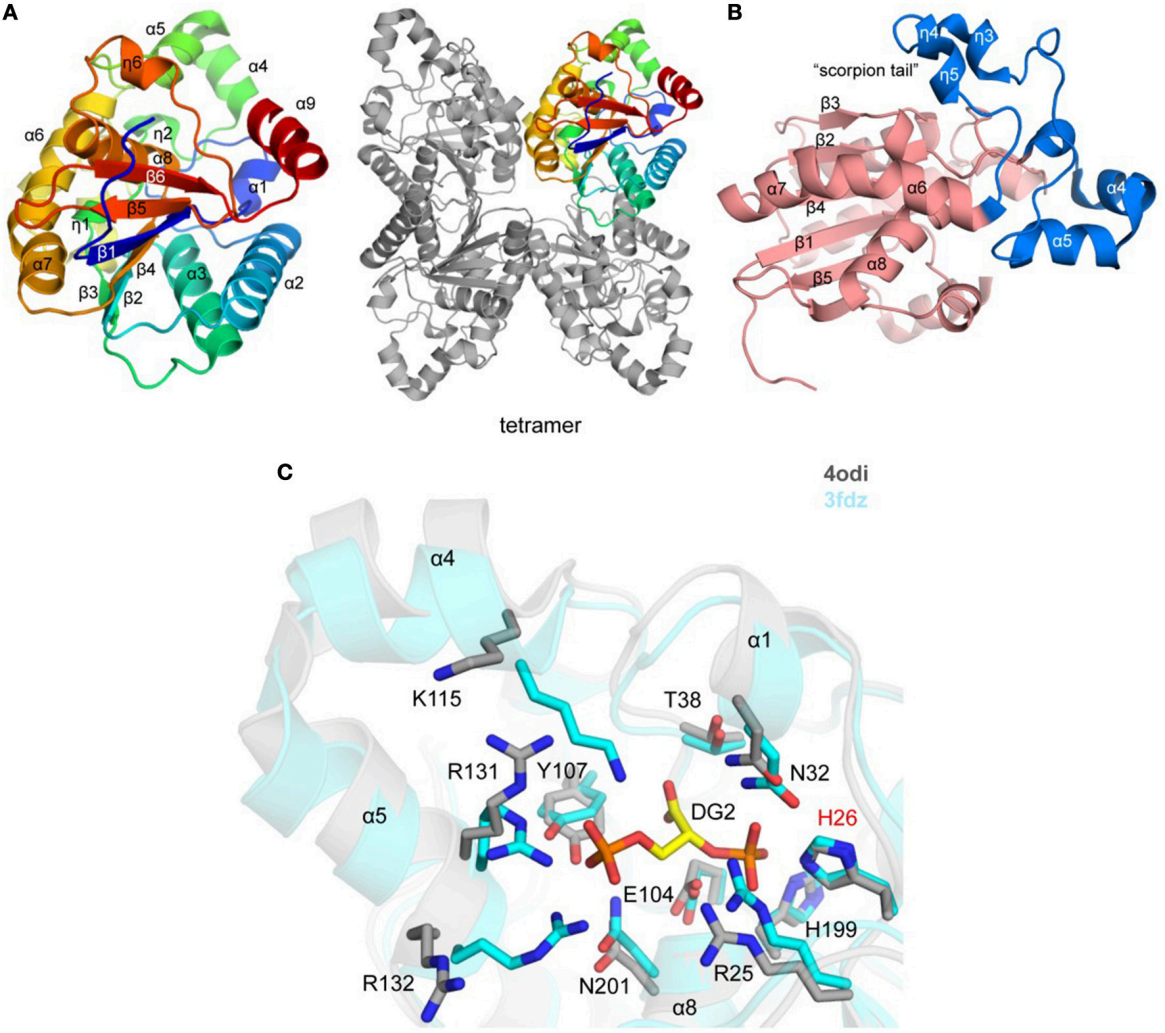
Crystal structure of *Tg*PGM **(A)**. Ribbon representation of *Tg*PGM monomer (left side) colored blue (N-terminus) to red (C-terminus) and tetramer of *Tg*PGM (right side) **(B)**. Domain structure of *Tg*PGM [red—an α/β/α-sandwich domain (residues 12–107 and 169–257) and blue—a domain without any defined folding motif (residues 108–168)] **(C)**. Pairwise structural alignment of *Tg*PGM (gray) and *Bp*PGM (cyan) showing active site with residues of *Tg*PGM shown in sticks and labeled in one-letter code. Equivalent residues of *Bp*PGM are displayed. *Bp*PGM binds (2R)-2,3-diphosphoglyceric acid, DG2, in the active site.

**Figure 3:**
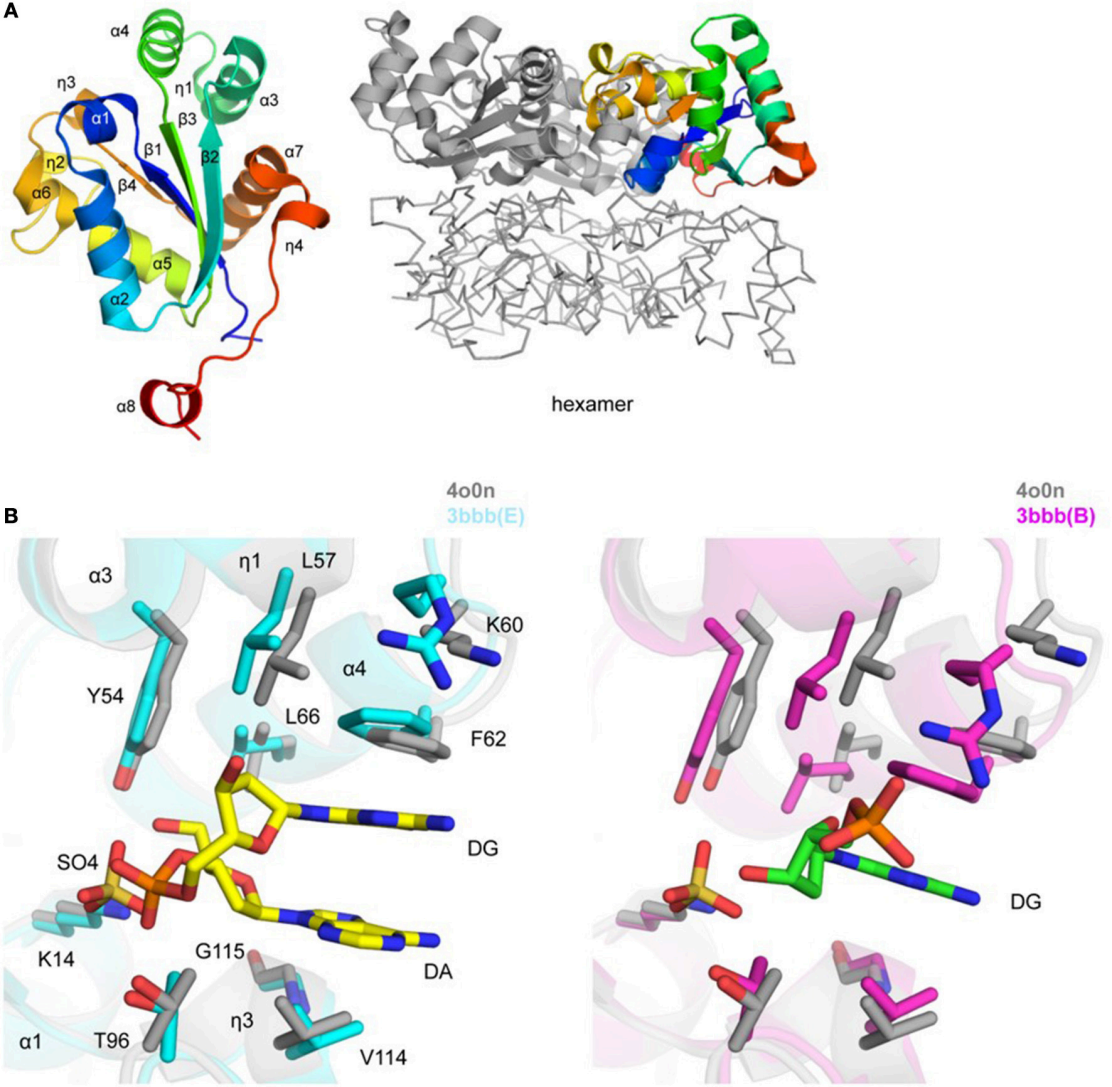
Crystal structure of *Tg*NDK **(A)**. Ribbon representation of *Tg*NDK monomer (left side) colored blue (N-terminus) to red (C-terminus) and trimeric and hexameric assemblies of *Tg*NDK (middle and right side) **(B)**. Pairwise structural alignment of *Tg*PGM (gray; SO4 is shown in sticks) and human NM23-H2 transcription factor [cyan (chain E with bound 2′-deoxyguanosine-5′-monophosphate (DG) and 2′-deoxyadenosine-5′-monophosphate (DA)] and magenta [chain B with bound 2′-deoxyguanosine-5′-monophosphate (DG)] showing active site with residues of *Tg*NDK shown in sticks and labeled in one-letter code. Equivalent residues of human NM23-H2 transcription factor are displayed.

**Figure 4:**
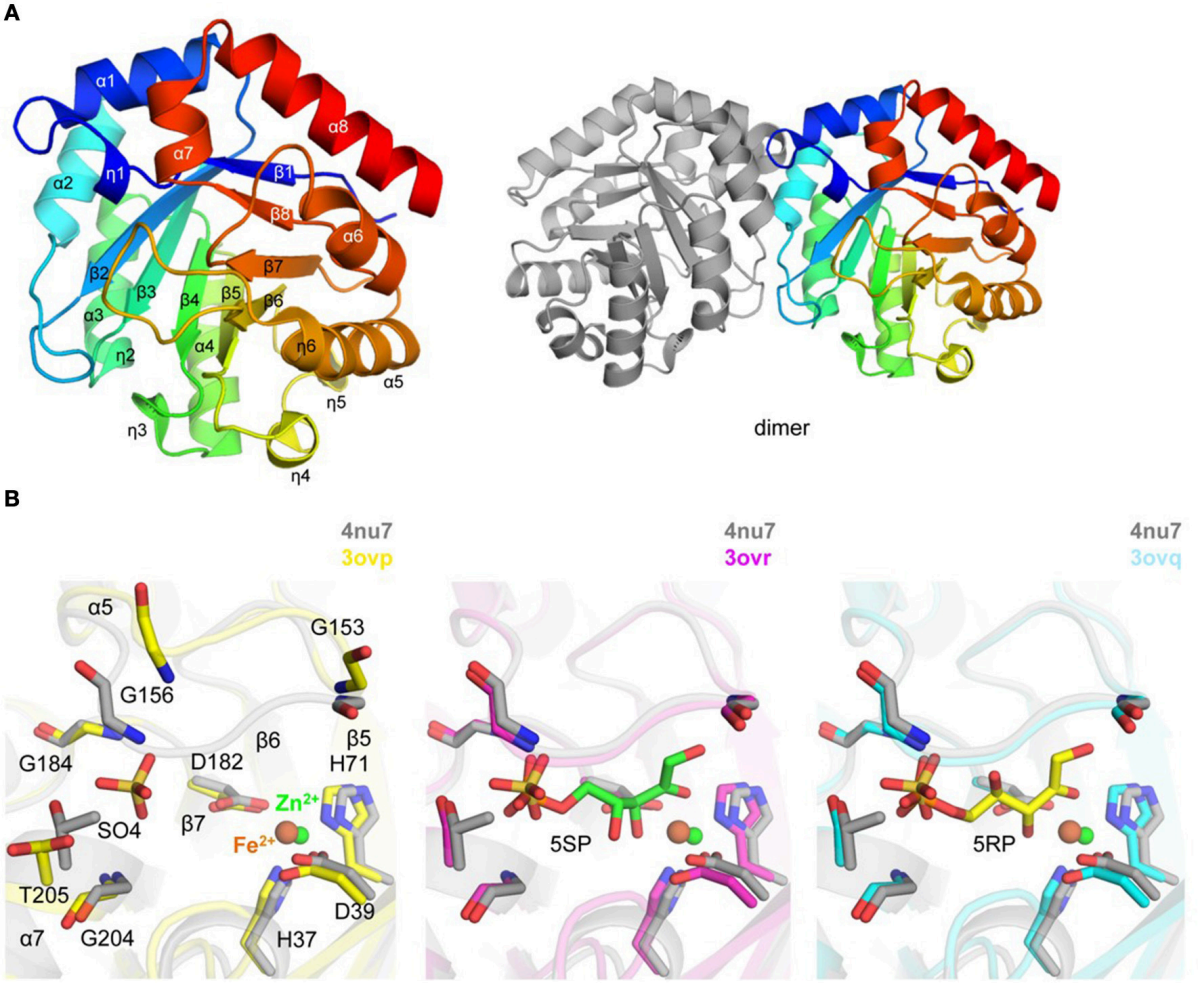
Crystal structure of *Tg*RPE **(A)**. Ribbon representation of *Tg*RPE monomer (left side) colored blue (N-terminus) to red (C-terminus) and *Tg*RPE dimer (right side) **(B)**. Pairwise structural alignment of *Tg*RPE (gray; SO4 is shown in sticks and Zn^2+^ as green sphere) and human RPE with bound Fe^2+^ (yellow ribbon), 5-O-phosphono-D-xylulose (5SR; magenta ribbon), and ribulose-5-phosphate (5RP; cyan ribbon) showing active site with residues of *Tg*RPE are shown in sticks and labeled in one-letter code. Equivalent residues of human RPE are displayed.

**Figure 5:**
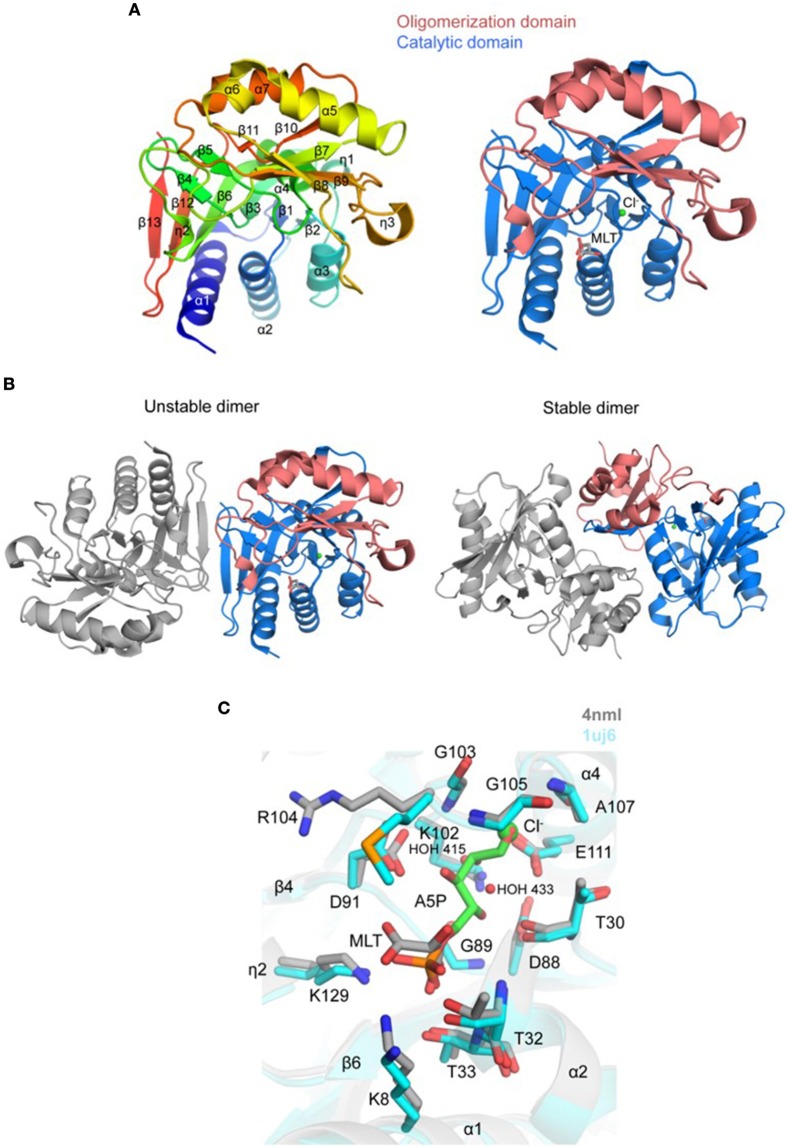
Crystal structure of *Tg*RPI **(A)**. Ribbon representation of *Tg*RPI monomer (left side) colored blue (N-terminus) to red (C-terminus). Catalytic domain and oligomerization domain of *Tg*RPI with bound D-malate (MLT; sticks) and chloride ion (Cl^−^; green sphere) are shown on the right. **(B)**. Unstable and stable *Tg*RPI dimers according to PISA prediction **(C)**. Pairwise structural alignment of *Tg*RPI (gray; MLT is shown in sticks and Cl^−^ as green sphere) and *Tt*RPI (cyan ribbon) with bound arabinose 5-phosphate (A5P; sticks) showing active site with residues of *Tg*RPI are shown in sticks and labeled in one-letter code. Equivalent residues of *Tt*RPI are displayed. Water molecules are shown as small red spheres.

**Figure 6:**
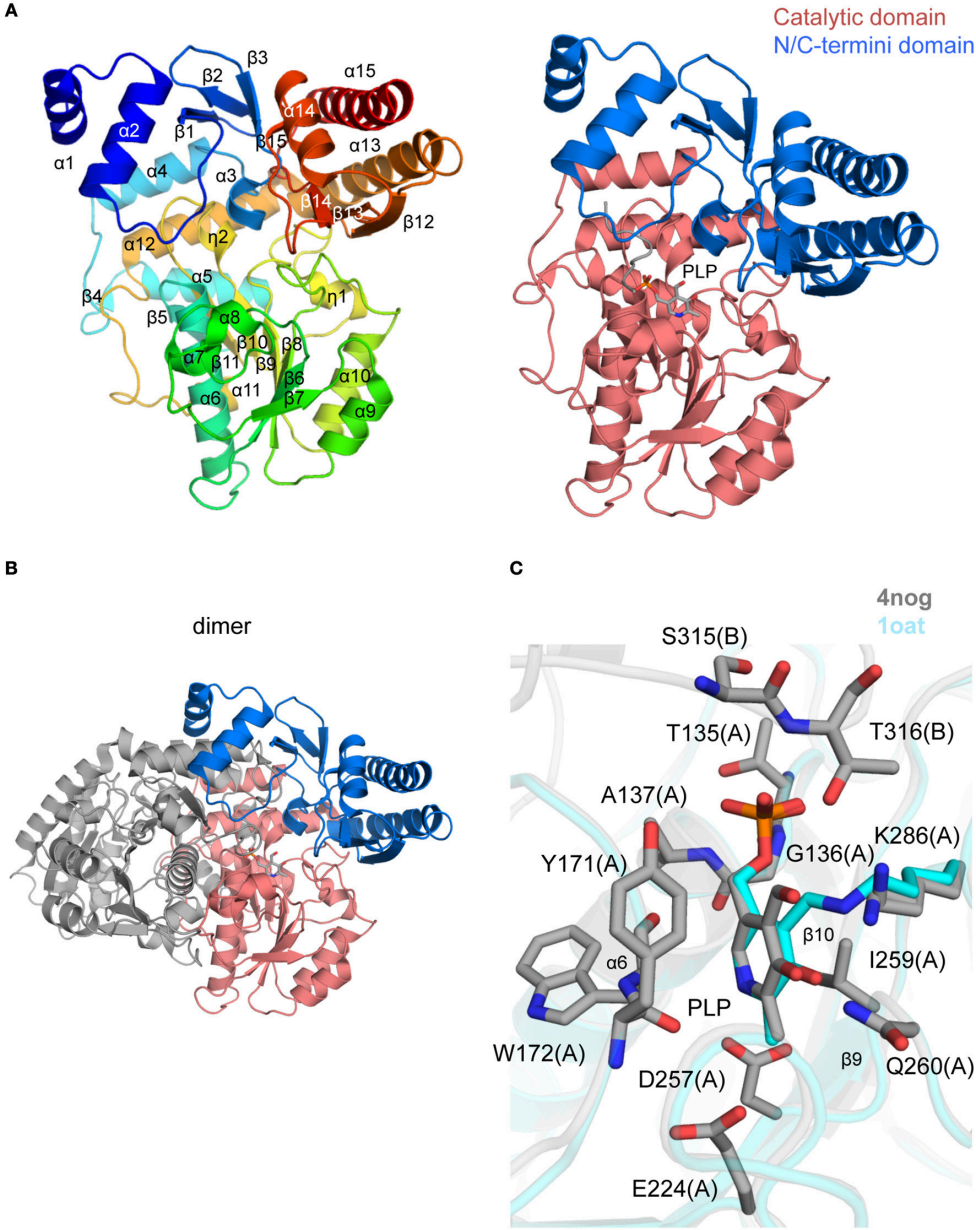
Crystal structure of *Tg*OAT **(A)**. Ribbon representation of *Tg*OAT monomer (left side) colored blue (N-terminus) to red (C-terminus). Catalytic and N/C-termini domains with bound PLP are shown on the right **(B)**. Dimer of *Tg*OAT **(C)**. Pairwise structural alignment of *Tg*OAT (gray; PLP is shown in sticks) and *h*OAT with bound PLP (cyan ribbon; PDB 1oat). Residues of *Tg*OAT are shown in sticks and labeled in one-letter code. Equivalent lysine residues of *h*OAT are displayed. Chain identifier in parentheses.

Each target protein was crystallized by the vapor-diffusion sitting-drop method mixing 1 μL of the protein in buffer A and 1 μL of a crystallization screen solution at 22°C. Single crystals were soaked in a crystallization condition for cryoprotection and flash frozen in liquid nitrogen for monochromatic X-ray data collection. Data were collected at 100 K from a single frozen crystal at the LS-CAT beamline 21-ID-F (λ = 0.97872 Å) at the Argonne National Laboratory (ANL), Advanced Photon Source (APS). Diffraction images were collected in oscillation mode and processed with *HKL*-3000 (Minor et al., 2006). Crystal structures of *Tg*PGM, *Tg*NDK, *Tg*RPE, and *Tg*OAT were determined by molecular replacement using *Phaser* (McCoy et al., 2007) from *CCP4* suite (Winn et al., 2011). Initial molecular replacement solutions were rebuilt with *ARP/wARP* (Morris et al., 2003). Crystal structure of *Tg*RPI was determined by single-wavelength anomalous dispersion (SAD) method in *HKL*-3000 (Minor et al., 2006). Non-Crystallographic Symmetry (NCS) restrains and Translation-Libration-Screw (TLS) groups refinement in *REFMAC* v.5.7 (Murshudov et al., 2011) were used to improve the quality of the structures. *Coot* (Emsley and Cowtan, 2004; Emsley et al., 2010) was used to manually check structures after each cycle of refinement in *REFMAC* and correct for side chain rotamers and fitting. The final models were validated with the Protein Data Bank (PDB) validation server (*ADIT* validation server; https://validate-rcsb-2.wwpdb.org/) and *MolProbity* (http://molprobity.biochem.duke.edu/) (Davis et al., 2007; Chen et al., 2010). Figures presenting crystal structures were prepared in the graphical program *PyMol* (The PyMOL Molecular Graphics System, Version 2.0 Schrödinger, LLC). Crystallization conditions, data-collection, structure determination and refinement statistics are summarized in Table 2. Crystal structures are described in Figures 2–6.

**Table 2:** Crystallization conditions, data-collection, structure determination and refinement statistics of *T. gondii* proteins.

**Protein name**	**PGM**	**NDK data set I**	**NDK data set II**	**RPE**	**RPI**	**OAT**
**CRYSTALLIZATION CONDITIONS**						
Screen conditions	0.2 M MgCl_2_, 0.1 M HEPES pH 7.5, 25% PEG3350	0.2 M ammonium sulfate, 0.1 M Bis-Tris pH 5.5, 25% PEG3350	0.2 M MgCl_2_, 0.1 M MES, 20% PEG6000	2 M ammonium sulfate, 0.1 M citric acid pH 3.5	2.1 M DL-Malic acid pH 7.0	0.2 M ammonium acetate, 0.1 M Bis-Tris pH 6.5, 25% PEG3350
Protein concentration (mg/ml)	7.5	7.5	6	7.4	7.1	22.8
**DATA COLLECTION**						
Space group	*P*2_1_2_1_2	*P*2_1_2_1_2_1_	*C2*	*H*32	*P4*_1_22	*P*1
Unit cell parameters (Å; °)	*a* = 97.2, *b* = 149.5, *c* = 72.1; α = β = γ = 90.0	*a* = 73.4, *b* = 121.6, *c* = 212.3; α = β = γ = 90.0	*a = 236.8, b = 73.4, c = 122.7; α* = γ = 90.0, β = 116.8	*a* = *b* = 138.5, *c* = 349.3; α = β = 90.0, γ = 120.0	*a* = *b* = 95.6, *c* = 112.7; α = β = γ = 90.0	*a* = 56.2, *b* = 61.3, *c* = 63.7; α = 100.6, β = 93.2, γ = 107.7
Resolution range (Å)	30.00–2.60 (2.64–2.60)	30.00–2.40 (2.44–2.40)	30.00-1.70 (1.73-1.70)	30.00–2.05 (2.09–2.05)	30.00–2.60 (2.64–2.60)	62.10–1.20 (1.22–1.20)
No. of reflections	33,033 (1,604)	75,092 (3,725)	202,729 (10,203)	81,012 (3,999)	16,774 (818)	224,574 (11,241)
*R*_merge_ (%)	10.6 (64.5)	8.4 (52.3)	5.9 (48.2)	7.2 (61.5)	9.4 (61.6)	4.3 (37.0)
Completeness (%)	100.0 (100.0)	99.6 (99.8)	98.5 (99.9)	100.0 (100.0)	100.0 (100.0)	91.0 (91.0)
〈*I*/σ(*I*)〉	18.3 (3.3)	18.2 (3.2)	22 (3.2)	20.1 (2.4)	44.9 (5.4)	11.2 (2.0)
Multiplicity	7.3 (7.4)	5.7 (5.8)	4.9 (4.9)	4.5 (4.5)	14.2 (14.7)	2.0 (2.0)
Wilson *B* factor	52.0	51.0	28.0	32.6	61.4	14.4
**STRUCTURE DETERMINATION**						
MR initial model (PDB ID)	1xq9	1ndl		3qc3	-	3lg0
**REFINEMENT**						
Resolution range (Å)	29.72–2.60 (2.67–2.60)	29.49–2.40 (2.46–2.40)	29.98-1.7 (1.74-1.7)	29.88–2.05 (2.10–2.05)	29.53–2.60 (2.67–2.60)	62.1–1.2 (1.23–1.20)
Completeness (%)	99.8 (98.2)	99.5 (99.7)	98.2 (97.04)	99.9 (99.9)	99.8 (99.8)	90.8 (90.0)
No. of reflections	31,317 (2,253)	71,019 (5,181)	192,440 (13,960)	76,865 (5,594)	15,802 (1,134)	213,305 (15,538)
*R*_work_/*R*_free_ (%)	19.7/23.9 (25.9/27.7)	19.2/23.8 (26.1/34.3)	15.4/19.2 (20.8/24.6)	14.9/18.7 (21.9/26.8)	16.4/20.5 (25.8/34.4)	13.3/16.5 (20.9/23.8)
Protein molecules/atoms	2/6,560	12/14,699	12/14,647	4/6,761	1/1,866	2/6,560
Solvent atoms	1,041	548	1854	513	71	1,041
Mean temperature factor (Å^2^)	56.3	48.7	33.4	39.9	56.8	18.4
**COORDINATE DEVIATIONS**						
R.m.s.d. bonds (Å)	0.011	0.007	0.010	0.010	0.012	0.021
R.m.s.d. angles (°)	1.626	1.219	1.398	1.434	1.711	1.927
**RAMACHANDRAN PLOT**^†^						
Most favored (%)	90.2	92.8	95.0	91.8	95.4	90.2
Allowed (%)	9.0	6.4	5.0	8.2	4.1	9.0
Generously allowed (%)	0.3	0.8	0.0	0.0	0.5	0.3
Outside allowed (%)	0.5	0.0	0.0	0.0	0.0	0.5
PDB Accession Code	4odi	4o0n	5bxi	4nu7	4nml	4nog

*Values in parentheses are for the highest resolution shell*.

† *Statistics are based on PROCHECK (Laskowski et al., 1993)*.

### Data accessibility

Coordinates and structure factors of the determined crystal structures were deposited to Protein Data Bank (www.rcsb.org) (Berman et al., 2000). Diffraction images for the deposited crystal structures can be found at the CSGID website (http://www.csgid.org/csgid/pages/home).

### Crystal structure data analysis

We used CATH/Gene3D v4.1 (http://www.cathdb.info/) (Sillitoe et al., 2015; Lam et al., 2016) to assess domain composition of studied proteins. Oligomeric state of the proteins and buried surface area (BSA) of multimers were estimated from crystal structure coordinates using PISA (http://www.ebi.ac.uk/pdbe/pisa/pistart.html) (Krissinel and Henrick, 2005, 2007; Krissinel, 2009). The number of intermolecular bonds that may contribute to protein quaternary structure was estimated from the structure coordinates using PDBsum (http://www.ebi.ac.uk/thornton-srv/databases/cgi-bin/pdbsum/GetPage.pl?pdbcode=index.html) (de Beer et al., 2014). Assessment of metal binding sites was done with help of the CheckMyMetal server (http://csgid.org/csgid/metal_sites) (Zheng et al., 2017). Protein structure comparison service PDBeFold at European Bioinformatics Institute (http://www.ebi.ac.uk/msd-srv/ssm) was used to identify structural homologs (Krissinel and Henrick, 2004). Pair-wise structural alignments were performed with DaliLite v.3 (https://www.ebi.ac.uk/Tools/structure/dalilite/) (Hasegawa and Holm, 2009).

### Molecular modeling

Schrödinger 2015-3, maestro v10.3 was used for binding site identification (SiteMap) on the five enzymes, and therefore, predict “druggability” by small molecules (Schrödinger Release 2015-3, 2018). A SiteScore threshold of 0.80 (with > 0.80 suggesting druggability) is used in this regard (Halgren, 2007).

### Cell culture

Human foreskin fibroblasts (HFF) were maintained in Iscove's Modified Dulbecco's Medium (IMDM) supplemented with 10% fetal bovine serum (FBS) and PENSTRA (penicillin and streptomycin). Cells were maintained at 37°C and 5% CO_2_.

### VivoPMO design

**Figure 7:**
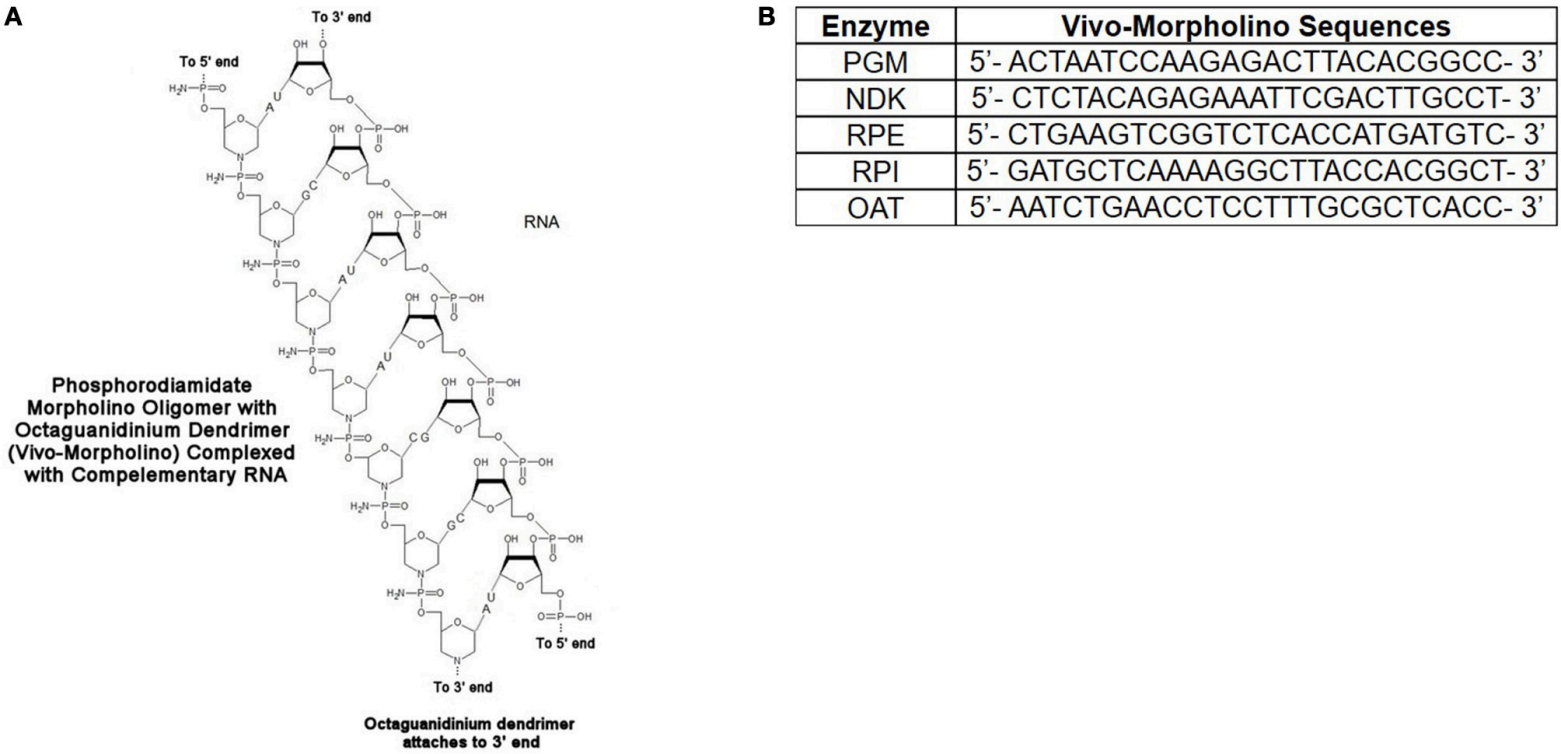
**(A)** Schematic of Interaction Between vivoPMO and RNA. **(B)** Sequences for PMO. Sequences for all enzymes were designed to be complementary to regions overlapping splice sites so as to prevent appropriate interaction between mRNA and the spliceosome predicted to lead to the production of a non-functional protein product.

The vivoPMOs were designed using genomic DNA sequences obtained from ToxoDB (accession numbers for genomic DNA sequences: PGM-TGME49_297060, NDK-TGME49_295350, RPE-TGME49_047670, RPI- TGME49_039310, OAT-TGME49_069110) with exon/intron junctions identified. One of these junctions was identified in each target gene and a vivoPMO was designed to be complementary to nucleotides on both sides of said junction. A diagrammatic representation of vivoPMO structure and RNA binding is in Figure 7A. The sequences of these morpholinos can be found in Figure 7B.

### VivoPMO efficacy assay

HFFs were grown in black, flat-bottomed 96-well microplates. HFFs were infected with 3,200 Type I RH parasites expressing yellow fluorescent protein (YFP). This allowed quantification of parasites *in vitro* post-treatment with vivoPMO. The parasites were incubated with the cells for 1 h, to allow sufficient time for invasion of HFFs, and were then treated with vivoPMO. Control triplicates with only fibroblasts and with pyrimethamine and sulfadiazine (the current standard of treatment for *T. gondii* infection) were also conducted. A concentration gradient of YFP parasites was also established, allowing quantification of knockdown. Several replicates of this efficacy assay were completed applying different concentrations of vivoPMO (2.5, 5, 10, and 20 μM). The cells and parasites were then incubated at 37°C for 72 h. This timing was previously established in other work. Fluorescence was measured using a Bio-Tek Synergy^TM^ H4 Hybrid Multi-Mode Microplate Reader. This methodology was consistent with previous work using morpholinos in *T. gondii* (Lai et al., 2012).

### VivoPMO toxicity assay

HFFs were grown in 96-well microplates, as in the efficacy assay. A gradient of dimethyl sulfoxide (DMSO) was used to quantify the amount of cell death caused by the vivoPMO *in vitro*. Different concentrations of vivoPMO (3.5, 5, 10, and 20 μM) were used to identify the level at which toxicity occurred. Following 72 h of incubation at 37°C, each well was treated with 10 μL WST-1, which reacts in metabolically active, viable cells through a complex set of chemical reactions dependent upon glycolytic NADPH production to form formazan dyes, which can be detected via a colorimeter. This method is also consistent with previous work (Lai et al., 2012).

### Data analysis of knockdown with vivoPMO

Knockdown was analyzed statistically using student *T*-test comparing parasite fluorescence between enzyme-specific vivoPMO and off-target vivoPMO. Additionally, the enzyme-specific vivoPMOs were compared to the levels of fluorescence at the standardized parasite load (2000 YFP-expressing parasites per well). Student *T*-test was also used to analyze toxicity data, comparing levels of optical density at 420 nm for untreated fibroblasts to treated cells. Statistical analysis was performed using STATA. Results were considered significant with *p* < 0.05.

### Antibody production

Antibodies were raised in mice at the University of Strathclyde (CR, SW). Briefly, mice were given two injections of formulated protein with NISV (non-ionic surfactant vesicle). The vesicles were made by melting mono-palmitoyl glycerol, cholesterol and dicetyl-phosphate (All from Sigma, UK) in a molar ratio of 5:4:1. 10 days after the final injection, serum was collected and tested by Western Blot using recombinant protein and *Toxoplasma* lysate.

### Immunofluorescence assay (IFA)

HFF cells were grown to confluence on sterilized coverslips in 24-well plates. Cells were fixed in 3% paraformaldehyde ~20–24 h after infection with Type I RH-strain tachyzoites and permeabilized in 0.25% Triton X-100. Serum from immunized mice, coupled with another primary antibody, RPS13, was applied at 1:500 dilution in PBS 1x/3% BSA/Triton-X-100 and detected using either Texas Red-conjugated goat anti-mouse antibody, or Alexa-488–conjugated goat anti-rabbit antibody. DAPI was used to stain for DNA. Coverslips were mounted with Antifade (Molecular Probes, Eugene, OR), and images were analyzed by high-resolution fluorescence using deconvolution protocols. Microscopy was performed with an inverted microscope (IX81; Olympus).

### Comparison to predicted essentiality via literature CRISPR/Cas9 screen

As an approach to identify whether target enzymes might be essential to parasite viability and fitness, a survey was done of phenotypic scores previously published in a genome-wide CRISPR/Cas9 screen (Sidik et al., 2016). Gene IDs were used and the average of the three available scores was taken. Negative scores were considered likely to be significant contributors to parasite fitness. Data of the genome-wide CRISPR/Cas9 screen are subsequently annotated in ToxoDB (http://toxodb.org/toxo/), including these gene IDs: *Tg*PGM: TGME49_297060, *Tg*NDK: TGME49_295350, *Tg*RPE: TGME49_247670, *Tg*RPI: TGME49_239310, *Tg*OAT: TGME49_269110.

## Results

### Structural genomic pipeline for *Toxoplasma*

The NIAID Structural Genomics Centers selected 265 proteins for the *Toxoplasma* Structural Genomic Pipeline in three phases (Figure 1). Phase I and II were a collaborative effort between CSGID and the *Toxoplasma* research community. Proteins were selected from published and unpublished work up to 2011. Selection was based on the mechanisms and pathways that are important for parasite infection and survival in human and animal hosts. Protein sequences were analyzed by XtalPred and final selection was also based on their crystallization feasibility (Slabinski et al., 2007).

Phase III utilized the TDR Database (Anderson, 2009; Crowther et al., 2010; Magariños et al., 2012). TDR integrated and weighed candidate drug targets based on extensive genetic, biochemical, pharmacologic, compound desirability and computationally-predicted druggability characteristics. Herein, 5 soluble enzymes were selected for further study. The genes encoding these enzymes were cloned, proteins expressed and purified, crystallized, and crystal structures were determined (Figures 2–6). The crystal structures have been deposited in the PDB database in accordance with CSGID and NIH policies. PDB codes can be found in Figure 1 and Table 2.

### Phosphoglycerate mutase II (PGM)

The crystal structure of a putative *Tg*PGM1 was determined and refined to 2.6 Å resolution (PDB 4odi) (Table 2 and Figure 2A). The *P*2_1_2_1_2 asymmetric unit comprises four PGM chains [~0.4 Å root-mean-square-deviation (r.m.s.d.) over 240 Cα atoms] assembled in two dimers with BSA of ~1,900 Å^2^, while four chains bury a surface area of ~ 6,800 Å^2^ (Figure 2A). Eleven salt bridges, 15 hydrogen-bonded and 236 van der Waals interactions hold the tetramer in place. A sodium ion-binding site (e.g., NA 301/A) was identified during structure refinement. Tyr155, Val158, and Asn160 coordinate NA 301/A. Residues 12–257 (chain A), 15–259 (chain B), 15–257 (chain C), and 17–248 (chain D) are present in the deposited structure.

*Tg*PGM has an α/β/α-sandwich domain that comprises residues 12–107 and 169–257 (Figure 2A). The domain has a six-stranded central β-sheet flanked by helices α1, α2, α3, α6, α7, α8, and α9, and 3_10_-helices η1 and η6. Residues 108–168 (helices α4 and α5, and four 3_10_ helices; η2–η5) flank the α/β/α domain without any defined folding motif (Figure 2B). Lys115 and Arg132 of this motif comprise a portion of the active site and together with Arg25, His26, Asn32, Thr38, Glu104, Tyr107, His199, and Asn201of the α/β/α domain shape the entrance to the active site (Figure 2C). An interesting feature of the structure is a “scorpion tail” segment (residues 136–168 belonging to 3_10_-helices η3, η4, and η5) that lies on top of the α/β/α domain (Figure 2B). Position of this segment within the crystal lattice suggests that it affects oligomerization and crystal packing. The tip of the tail possesses the sodium ion-binding site.

The structure of the *Plasmodium falciparum* PGM (*Pf* PGM; PDB 3kkk; DOI: 10.2210/pdb3KKK/pdb) is the closest homolog of *Tg*PGM with estimated 75% sequence identity and ~0.6 Å r.m.s.d. over 229 Cα atoms. The structure of the homologous bacterial enzyme from *Burkholderia pseudomallei* (PDB 3fdz; 67% sequence identity; r.m.s.d. ~1.0 Å over 230 Cα atoms) determined in the complex with (2R)-2,3-diphosphoglyceric reveals that *Tg*PGM possesses identical active site residues with an essential phospho-acceptor residue His26 (Davies et al., 2011). Preliminary validation of this active site in Schrödinger SiteMap gave a favorable score of 0.98. Structural alignment of *Bp*PGM and *Tg*PGM demonstrates significant main- and side-chain conformational changes at the protein active site that would most likely take place in *Tg*PGM during the interconversion of its substrate 3-phosphoglycerate to 2-phosphoglycerate (Figure 2C). Conformational differences were also observed for residues located on the tip of the scorpion-like tail.

### Nucleoside diphosphate kinase (NDK)

Crystal structures of a putative *Tg*NDK were determined and refined to 2.4 Å (PDB 4o0n) and 1.7 Å (PDB 5bxi) resolution (Figure 3A and Table 2). Monomers in both *Tg*NDK structures are superimposed with ~0.2–0.7 Å r.m.s.d. over 153 Cα atoms, while monomers in 4o0n align with ~0.2–0.7 Å r.m.s.d (153 Cα atoms) and monomers in 5bxi align with ~0.2–0.6 Å r.m.s.d (153 Cα atoms). In both structures, *Tg*NDK forms a hexamer with an average BSA of ~18,900 Å^2^ (Figure 3A). Comparison of *Tg*NDK with known crystal structures of homologs proteins suggests that the *Tg*NDK hexamer is likely a biologically functional assembly (Min et al., 2002; Vieira et al., 2015).

Residues 156–160 (GENLY) of the C-terminal tag in chains D and G only of the 5bxi structure were modeled, while they are absent in all chains of the 4o0n structure. These residues protrude to the solvent and make contacts with symmetry-related hexamer. The side chain of Tyr160 is spatially positioned in a nucleotide base-binding pocket of the active site of a symmetry-related molecule (not shown). In addition, residues 56–64 (DLKGKPFFP; chain C of 5bxi) that belong to the C-terminus of helix α3, 3_10_ helix η1, η1–α4 loop, and the N-terminus of helix α4 are disordered. This peptide stretch may constitute a portion of a nucleotide base-binding pocket of the active site. Superposition of all twelve chains in 5bxi and 4o0n structures revealed that this segment has similar secondary structure conformations. Presumably, lack of favorable crystal contacts resulted in its disorder in chain C of the 5bxi structure.

*Tg*NDK adopts an α/β/α sandwich fold with four β strands and eight surrounding α helices (Figure 3A). The putative active site of *Tg*NDK was identified based on a pairwise structural alignment with the crystal structure of human NM23-H2 transcription factor in complex with the dinucleotide d(AG) (PDB 3bbb; Dexheimer et al., 2009). The active site comprises residues Lys14, Tyr54, Leu57, Lys60, Phe62, Leu66, Thr96, Val114, and Gly115 (Figure 3B), where analysis by Schrödinger SiteMap revealed a score of 0.99 (using PDB 4o0n), indicating a “druggable” pocket. Crystals of *Tg*NDK (PDB 4o0n) grew in the presence of ammonium sulfate and, thus, several sulfate ions were identified and modeled during refinement. Superposition of *Tg*NDK with the structure of human NM23-H2 transcription factor (~67% homology; ~0.6 Å r.m.s.d. over 148 Cα atoms) revealed that a sulfate ion (e.g., SO4 201/A) binds close to a phosphate-binding pocket of the active site (Figure 3B). A bicarbonate ion (e.g., BCT 201/A) was modeled at a similar location in the 5bxi structure. Similar main- and side-chain rearrangements of residues of the active site as seen in NM23-H2 are expected in *Tg*NDK upon binding its substrate.

### Ribulose phosphate 3-epimerase (RPE)

Crystal structure of *Tg*RPE was determined and refined to 2.05 Å resolution (PDB 4nu7; Table 2 and Figure 4A). *Tg*RPE adopts an aldolase-type TIM-barrel α/β fold resembling homologous RPEs. Four chains of *Tg*RPE (~0.3–0.5 Å r.m.s.d. over 225 Cα atoms) in the *H*32 asymmetric unit form two dimers (A–C and B–D; Figure 4A) each burying a surface area of ~3,500 Å^2^. The dimer is similar to one observed for known RPEs (Williamson and Wood, 1966; Jelakovic et al., 2003; Caruthers et al., 2005; Liang et al., 2011). Helices α1 and α2, and the β2–α2 and η1– α1 loops of *Tg*RPE comprise the dimer interface with 4 salt bridges, 8 hydrogen-bonded and 130 van der Waals contacts (e.g., between chains A and C). PISA prediction and crystal packing analysis revealed a hexameric assembly of *Tg*RPE (BSA is ~13,300 Å^2^; not shown) that has been reported previously for homologous RPEs (Kopp et al., 1999; Wise et al., 2004; Akana et al., 2006).

A Zn^2+^ (e.g., ZN 303/A) ion was modeled per each chain of *Tg*RPE, with His37, Asp39, His71, Asp182 and a water molecule coordinating the metal (Figure 4B). Similar coordination has been reported for D-xylulose 5-phosphate co-crystallized with human RPE enzyme (PDB: 3ovr) (Liang et al., 2011) (Figure 4B). PDBeFold identified human RPE as the closest homolog of *Tg*RPE (~0.9 Å r.m.s.d. over 215 Cα atoms; 52% sequence identity). The CheckMyMetal analysis identified that Co^2+^ and Cu^2+^ may also bind in a similar position as zinc in *Tg*RPE. It is known that the RPE enzymes can utilize Fe^2+^, Co^2+^, and Mn^2+^ for catalysis (Jelakovic et al., 2003; Wise et al., 2004; Caruthers et al., 2005; Akana et al., 2006; Liang et al., 2011). Residues of the metal binding site are strictly conserved in the RPE enzymes. Structural superposition revealed that Fe^2+^ in ferrous-, substrate- and product-bound human RPE structures is ~1.0 Å away from the position of Zn^2+^ in *Tg*RPE. We also compared *Tg*RPE structure with two other RPEs structures (PDBs 1tqx and 1h1z) (Jelakovic et al., 2003; Caruthers et al., 2005) that have zinc and ${\text{SO}}_{4}^{2-}$ bound in similar locations as *Tg*RPE. Positions of zinc and side chains of the Zn^2+^-coordinating residues are similar in the three compared structures (not shown). Other RPE structures with bound Zn^2+^ and other than ${\text{SO}}_{4}^{2-}$ ligands (e.g., PDBs 2fli, 3qc3, and 5umf) have similar positions of Zn^2+^ and side chains of the metal-binding residues (Akana et al., 2006; Joint Center for Structural Genomics, 2011; Dranow et al., 2017). Additional experiments are needed to confirm the biological importance of zinc in the *Tg*RPE-based catalysis. This metal-binding region was analyzed for “druggability” and scored 1.04 (Schrödinger SiteMap).

Crystals of *Tg*RPE grew in the presence of 500 mM NaCl and 2 M ammonium sulfate and, thus multiple sulfate and chloride ions were modeled to interpret additional electron density. Structure comparison analysis revealed that a sulfate ion (e.g., SO4 304/A) in *Tg*RPE occupies the binding site of a phosphate moiety of substrate or product observed in the structure of the human RPE enzyme (PDBs 3ovq and 3ovr) (Figure 4B). In all ligand-bound human RPE structures, a loop (e.g., the β6–α5 loop in *Tg*RPE) supports position of a ligand at the active site, i.e., moves from its ligand-free conformation to cap a ligand bound to the enzyme. Thus, similar loop movement is expected for *Tg*RPE upon binding of the substrate, i.e., a ligand. Apparently, binding of ${\text{SO}}_{4}^{2-}$ sufficed the loop repositioning in *Tg*RPE (Figure 4B).

### Ribose 5-phosphate isomerase (RPI)

Crystal structure of a putative *Tg*RPI was determined and refined to 2.6 Å resolution with one protein molecule per asymmetric unit (PDB 4nml; Figure 5A and Table 2). *Tg*RPI has a larger α/β/α sandwich catalytic domain (residues 1–127 and residues 225–259) with the Rossmann topology and a smaller α/β sandwich oligomerization domain (residues 128–224) (Figure 5A). Residues 180–186 between strand β8 and a 3_10_-helix (η3), Arg258, and Lys259 are absent in the structure due to their disorder. BME was used in buffers during purification and crystallization and is covalently linked to Cys82 in the structure. One D-malate molecule and two chloride ions were modeled to interpret additional electron density (Figures 5A,C). Applying crystal symmetry operations PISA predicted that *Tg*RPI might exist as unstable dimer (BSA of ~2,430 Å^2^) or stable dimer (BSA of ~3,340 Å^2^) in the crystal environment (Figure 5B). Visualization of both dimers revealed that the first assembly is held in place primarily by hydrogen-bonded interactions between main-chain atoms of strand β13 of each monomer's catalytic domain (Figure 5B). The second dimer is stabilized by multiple intramolecular contacts between residues from both *Tg*RPI domains. Homologous RPIs form similar stable dimers and, thus stable *Tg*RPI dimer may be considered biologically relevant.

PDBeFold identified several homologs of *Tg*RPI, and we used the crystal structure of RPI from *Thermus thermophilus* (*Tt*RPI; PDB 1uj6) (Hamada et al., 2003) determined in complex with arabinose 5-phosphate to identify the active site of *Tg*RPI (Figure 5C). Both proteins share 45% sequence homology and are superimposed with ~1.0 Å r.m.s.d. over 218 Cα atoms. This structural alignment revealed that D-malate and chloride ion (e.g., CL 301/A) in *Tg*RPI are bound in the protein's active site, mimicking parts of arabinose 5-phosphate in 1uj6 (Figure 5C). D-malate makes hydrogen bonds with side chains of Lys8, Thr32, and Thr33, and several van der Waals contacts with residues of the phosphate-binding pocket of the active site (Schrödinger SiteMap score 0.97). CL 301/A is bound in the oxyanion hole of the active site and coordinated by main-chain nitrogen atoms of Gly105 and Ala107, side chain carboxyl group of Glu111, and two water molecules, HOH 415 and HOH 433 (Figure 5C). The second chloride ion (e.g., CL 302/A) in *Tg*RPI binds near disordered residues 179–185 and makes bonds with main-chain nitrogen atoms of Phe172 and Ile190, and the ε-amino group of Lys154 (not shown).

The catalytic domain of homologous RPIs align well, while the oligomerization domain has some distinct structural differences among various species. For example, a peptide stretch between residues Leu160 and Arg169 of the oligomerization domain of *Tg*RPI is longer than the equivalent region in *Tt*RPI. In the *Tg*RPI, structure this segment is helix α6. We have found that a similar helical element is present in *E. coli* RPI (Zhang et al., 2003) (PDB 1o8b; 45% sequence homology; ~1.4 Å r.m.s.d. over 164 Cα atoms) and *P. falciparum* RPI (Holmes et al., 2006) (PDB 2f8m; 45% sequence homology; ~1.1 Å r.m.s.d. over 233 Cα atoms). Another example, strands β8 and β9 in *Tg*RPI are separated by a 3_10_-helix (η3) and disordered residues 179–185, while similar region in *Tt*RPI, *Ec*RPI, and *Pf* RPI is shorter, ordered, and extends toward the N-terminus of helix α4 of the superimposed *Tg*RPI. Thus, η3 and residues 179–185 seem to be unique to *Tg*RPI. Its length, proximity to the phosphate-binding pocket, and potential flexibility suggest that it may participate in the catalysis.

### Ornithine aminotransferase (OAT)

The crystal structure of *Tg*OAT in complex with pyrodoxal-5′-phosphate (PLP) was determined at 1.2 Å resolution (PDB 4nog; Figure 6A and Table 2) and contains two protein chains in the asymmetric unit (~0.3 Å r.m.s.d. over 422 Cα atoms) that form a well-studied functional dimer with BSA of ~5,770 Å^2^ (Shah et al., 1997; Shen et al., 1998; Markova et al., 2005; Vedadi et al., 2007; Jortzik et al., 2010) (Figure 6B). Each monomer has a mixed α/β structural fold and consists of the cofactor PLP-binding domain (residues 87–336), an N-terminal domain (residues 17–86), and a C-terminal domain (residues 337–441) (Figure 6A). The PLP-binding domain comprises the eight-stranded central β-sheet linked to 10 α-helices and 4 short α-helical segments. The N- and C-terminal domain comprises three α-helices bounded to a β-sheet of three and four β strands, respectively. The difference between *Tg*OAT monomers is observed at the position of N-terminal α-helix and is likely affected by crystal packing. BME used in the protein buffer solutions forms a covalent bond to Cys353 in both *Tg*OAT monomers. One 1,3,5-*Tris*(4-carboxyphenyl)benzene (BTB), polyethylene glycol (PEG) and acetate (ACT) molecule were modeled to interpret additional electron density in chain B.

The dimerization interface is primarily formed between residues of the PLP-binding and N-terminal domains of *Tg*OAT. The dimer is held by 3 salt bridges, Cys96–Cys96 disulfide bond, 63 hydrogen bonds and 782 van der Waals interactions. The *Tg*OAT dimer bears structural similarities to homologous enzymes from the aminotransferase class-III protein family (Christen and Metzler, 1985; Mehta et al., 1993) and, thus, may be considered biologically relevant. The list of homologous structures with sequence identity >40% encloses structure of OAT from human (r.m.s.d. ~0.9 Å over 404 Cα atoms) (Shah et al., 1997; Storici et al., 1999), OAT from *P. falciparum* (r.m.s.d. ~1.2 Å over 384 Cα atoms) (Vedadi et al., 2007; Jortzik et al., 2010) and OAT from *P. yoelii* (r.m.s.d. ~1.0 Å over 369 Cα atoms) (Vedadi et al., 2007). Among structures with similar secondary structure fold and high r.m.s.d. value (~1.9 Å over 395 Cα atoms) is the structure of GABA-OAT from *E. coli* with sequence identity 31%. Recent reports indicate that *Tg*OAT shows a dual N-acetyl-ornithine (AcOrn) and γ-aminobutyric acid (GABA) transaminase activity and may function in both arginine and GABA metabolism (Astegno et al., 2017).

The cofactor-binding domain of *Tg*OAT non-covalently binds PLP molecule, i.e., PLP does not form the Schiff base with conserve Lys286 (Figures 6A,C). Similarly to homologous OAT structures obtained in complex with the cofactor (Shah et al., 1997; Shen et al., 1998; Storici et al., 1999; Vedadi et al., 2007; Jortzik et al., 2010), PLP interacts with Thr135, Gly136, Ala137, Tyr171, Trp172, Glu224, Asp257, Ile259, Gln260, Lys286, Ser315, and Thr316 (Figure 6C). Most of these residues are highly conserved among *Tg*OAT homologs and represent a viable, “druggable” pocket (SiteMap score 1.03).

### *In vitro* studies of targeted vivoPMO on *T. gondii* replication

**Figure 8:**
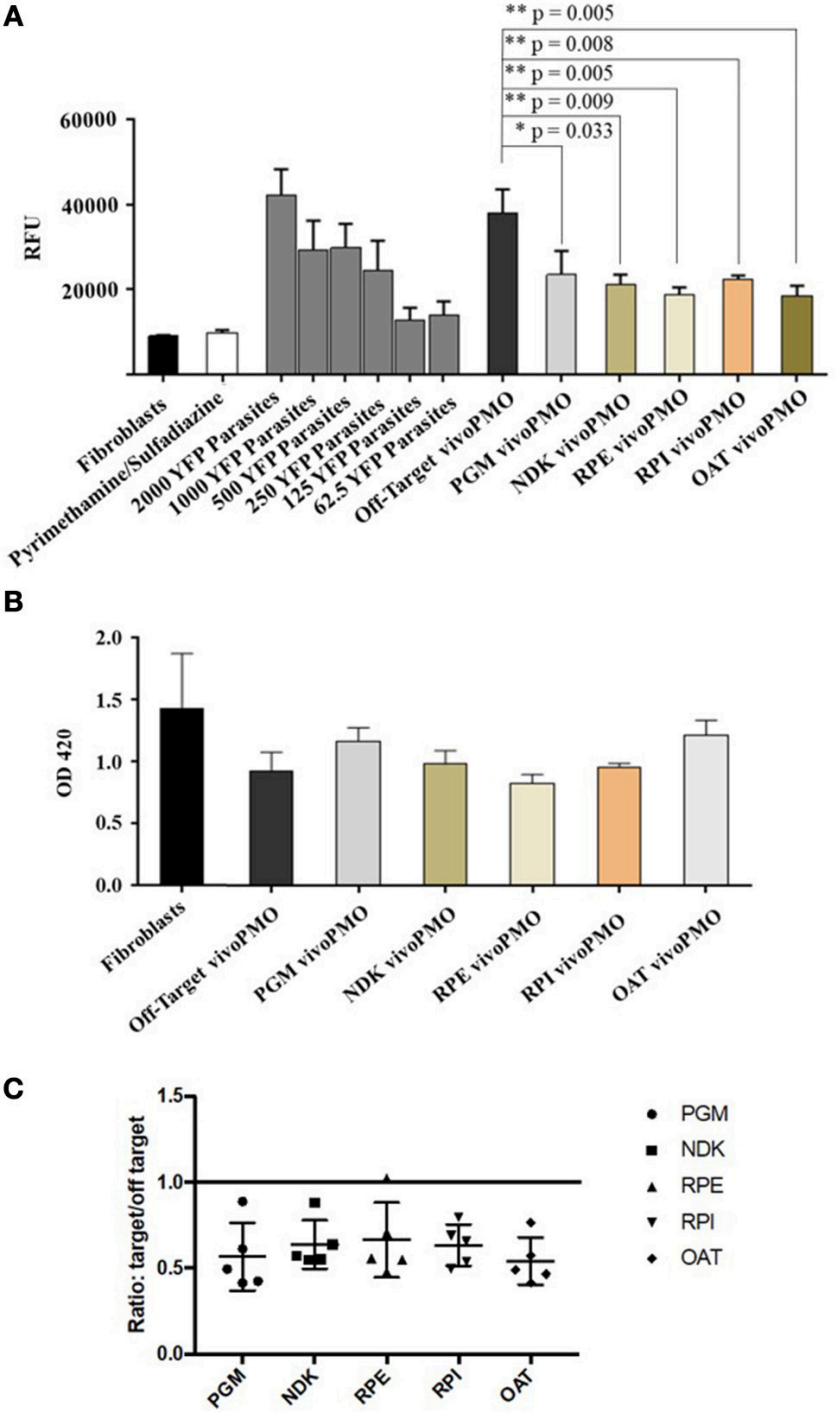
**(A)** YFP/vivoPMO Efficacy Assay at 10 μM. Targeted vivoPMOs show statistically significant knockdown relative to off-target and to a standardized parasite concentration as determined by student *t*-test. **(B)** Wst-1 Cell Viability/ vivoPMO Toxicity Assay at 10 μM. Targeted vivoPMOs do not show statistically significantly (*p* < 0.05 by student *T*-test) different levels of optical density relative to off-target vivoPMO or uninfected fibroblasts at 10 μM concentrations. **(C)** Ratio of On- to Off-Target vivoPMO for Replicate Experiments Using 10 μM of Targeted vivoPMO. The data presented here represent five replicate experiments. Lower individual values suggest a larger effect of the morpholino.

HFFs were infected with YFP-expressing *T. gondii* tachyzoites and treated with vivoPMOs targeted against all five enzymes of interest. When treated at 10 μM concentrations, each morpholino resulted in approximately 50% reduction in fluorescence *in vitro* (between 44% for PGM-targeted vivoPMO to 56% reduction for OAT-targeted vivoPMO). This was compared to the off-target, control morpholino, which resulted in < 10% fluorescence reduction. When compared statistically using student *T*-test, levels of fluorescence *in vitro* were statistically decreased (*p* < 0.05) when treating with targeted vivoPMO relative to untreated fibroblasts infected with 2000 YFP-expressing tachyzoites as well as when compared to infected cells treated with off-target morpholino. A representative experiment showing these findings is in Figure 8A. Data from all replicate experiments of the morpholino efficacy assays, as well as statistical calculations and ratios reflecting efficacy of targeted morpholino to off-target can be found in Supplementary Table 1.

To confirm that these findings of decreased fluorescence *in vitro* was not secondary to host-cell toxicity from the vivoPMOs, a WST-1 assay was used. HFFs treated with 10 μM concentrations of enzyme-targeted vivoPMOs were statistically indistinguishable (*p* > 0.05) from untreated cells by Student *T*-test. Toxicity was demonstrated at higher vivoPMO concentrations of 20 μM. A representative experiment demonstrating the absence of host-cell toxicity with vivoPMO treatment at 10 μM is in Figure 8B.

The ratio of targeted to off-target vivoPMO are displayed in Figure 8C. This is shown for five replicate experiments (Supplementary Table 1). This demonstrates that, in cultures treated with on-target vivoPMO, parasitic replication was reduced.

These findings were compared with the effect on tachyzoites in a previously published full genome screen using CRISPR/Cas9 (Sidik et al., 2016). Three of the target proteins (*Tg*PGM, *Tg*RPE, and *Tg*RPI) that had a significantly reduced replication phenotype in tachyzoites caused by vivoPMO knockdown were found to contribute to parasite fitness when studied using CRISPR/Cas9. The CRISPR/Cas9 data was annotated and graphically presented in ToxoTB (http://toxodb.org/toxo). *Tg*PGM, *Tg*RPE, and *Tg*RPI show negative phenotypic/fitness scores (−4.45, −0.31, −1.92, respectively) suggesting essentiality. *Tg*OAT appeared dispensable (fitness score of +1.2) in the tachyzoite CRISPR/Cas9 assays consistent with *Tg*OAT appeared to be expressed predominantly in the oocyst stage (ToxoDB). *Tg*NDK (+2.45) also appeared to be dispensable in tachyzoites.

### Immunofluorescence assay

**Figure 9:**
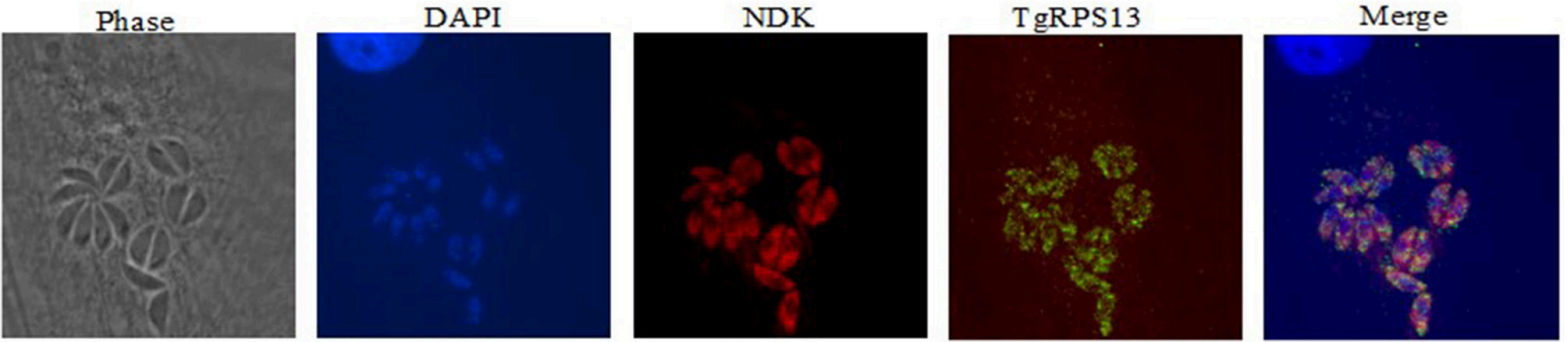
Immunofluorescence Assay. This series of images depict the localization of nucleoside diphosphate kinase, one of the molecular targets herein discussed. Note the concentration of red fluorescence at the periphery of the parasite. This is consistent with the localization of secreted proteins like Gra1, suggesting that NDK could be secreted by the parasite. This has precedents in other pathogens, including *M. tuberculosis* and *Leishmania*.

Recombinant protein for the five targets was also used to produce antibodies in mice, which were used for immunostaining to determine expression patterns within the parasite. Immunolocalization was not successful with tachyzoites of *Toxoplasma* for four of the five enzymes. For NDK, enzyme was present in a granular pattern in the cytoplasm of tachyzoites, located around the perimeter of the parasite and the posterior part of the parasite. This can be seen in Figure 9.

## Discussion

**Figure 10:**
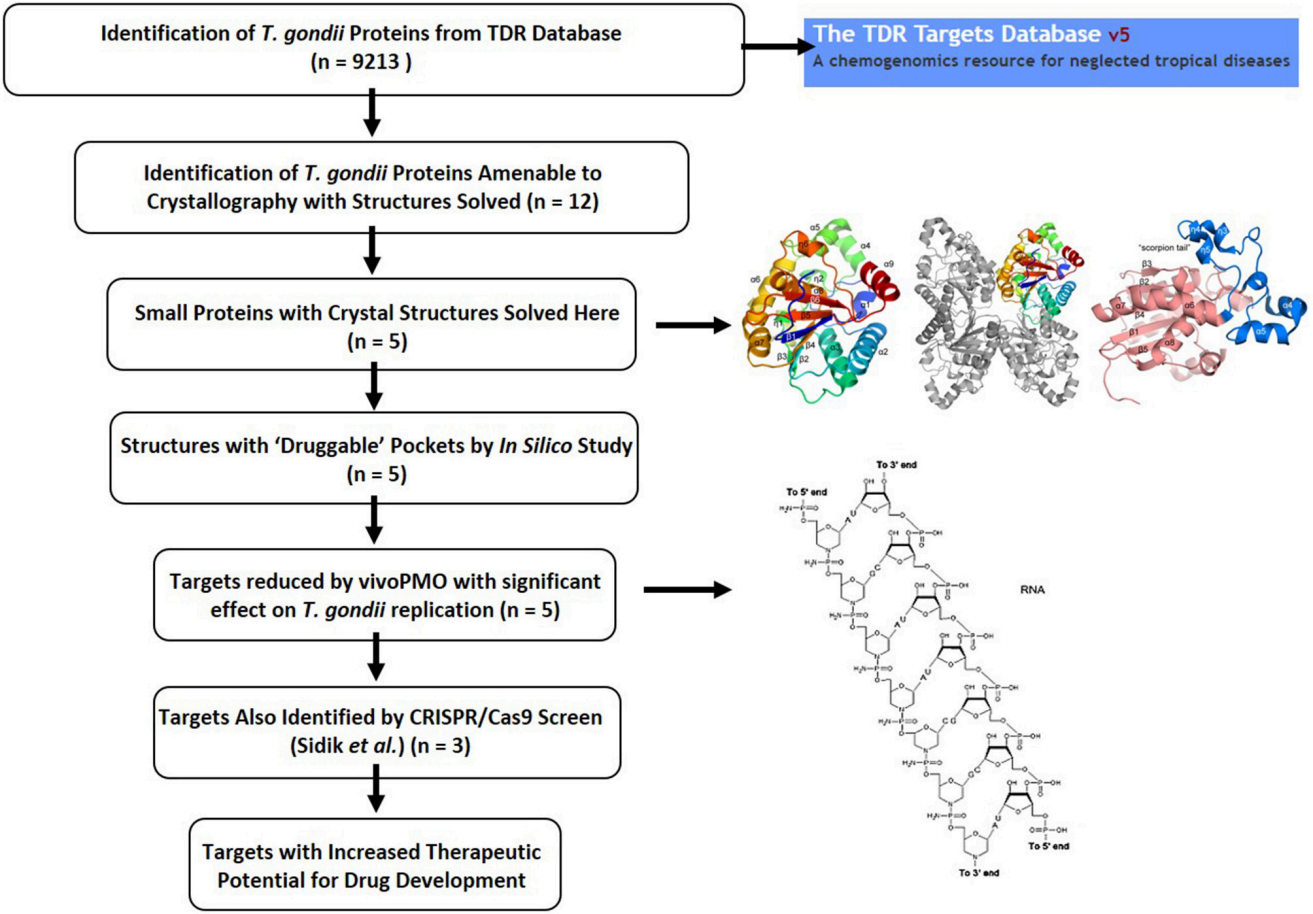
Summary figure showing the *T. gondii* drug discovery pipeline as described herein. The TDR Database provided important initial insights into potentially important parasite proteins (Agüero et al., 2008; Magariños et al., 2012). Identification of proteins amenable to crystallographic analysis, solution of their structures, abrogation with vivoPMO and confirmation of essentiality by querying the CRISPR/Cas9 database completed the pathway, identifying enriched targets with improved therapeutic potential.

The work characterized herein presents a detailed characterization of enzyme structure that can be used for modeling inhibitors of these targets and also presents approaches for studying target phenotype with vivoPMO and CRISPR/Cas9 which in combination develops a system to move forward candidate targets (Figure 10). The targeted vivoPMOs demonstrated statistically significant perturbation of parasitic replication when compared to off-target morpholinos, without concomitant host-cell toxicity, confirmed for some of these with CRISPR/CAS9 screen. Inhibition of target enzymatic function via small molecules or anti-sense might be a novel therapeutic modality, should such small molecules exist. Given the current limitations of anti-parasitic medicines for the treatment of toxoplasmosis, new pharmacotherapy is of significant interest. The multi-step approach, detailed here, of *in vitro* inhibition through anti-sense techniques accompanied by detailed structural characterization to identify possible exploitable differences between host and parasite enzymes. Confirmation by referring to a recently published CRISPR/Cas9-based analysis, can also help to suggest importance of targets in *T. gondii* tachyzoites (Sidik et al., 2016). This provides another level of evidence that particular targets may be important for the parasites replication. This approach was performed for each of five enzymes identified as having potential biologic importance and with favorable predicted druggability using the TDR methodology. This favorable druggability was also confirmed with *in silico* modeling using Schrödinger SiteMap, which indicated each target demonstrated “druggable” pockets. Each enzyme will be considered sequentially hereafter.

Phosphoglycerate mutase II, an enzyme at the core of the glycolytic pathway, catalyzes the transition from 3-phosphoglycerate to 2-phosphoglycerate, an important preparatory step upstream of enolase and pyruvate kinase. Although little work had been done on this Apicomplexan enzyme as a potential drug target, we inferred that interrupting glycolysis would prevent the production of pyruvate by glycolysis, and thereby reduce input into the Kreb's Cycle. This, in turn, will reduce the number of electron carrying compounds (NADH, FADH_2_), which would markedly reduce ATP production. Interestingly, glycolysis has been targeted successfully in other parasites, and also has been demonstrated to have importance to host cell egress, as well as maintenance of energy reserves when the parasite is found outside of host cells (Ananvoranich et al., 2006; Fleige et al., 2007; Pomel et al., 2008; Lin et al., 2011; Singh et al., 2013). These observations, coupled with our own, are consistent with the observed effect on parasite replication. It appears that putative inhibitors could make this a robust tachyzoite molecular target with a compound easily available to test. With the present crystal structure, further structure-based molecular design approaches (such as virtual screening) is possible given the favorable SiteMap score (0.98).

Nucleotide diphosphate kinase catalyzes the movement of phosphate from a nucleoside triphosphate to a nucleoside diphosphate (e.g., GTP + ADP –> GDP + ATP). Naturally, disruption of this process could have an impact on the energy economy within the parasite, as ATP would not be available for important cellular tasks related to DNA replication and the production of more parasites. Additionally, this is a stress kinase, and thus its impact on the stressed organism (through a tetracycline-dependent gene expression construct, RPS-13) would be useful to explore. An immunofluorescence assay revealed peripheral and posterior concentration of staining. Interestingly, another intracellular pathogen, *Leishmania amazonensis*, has been shown to secrete NDK to prevent host-cell autolysis (Kolli et al., 2008). It would be of interest to determine whether this enzyme plays a similar role in Apicomplexans like *T. gondii*. Of note, NDK inhibition by candidate compounds has been demonstrated to have efficacy against certain species of *Leishmania in vitro* (Vieira et al., 2015; Mishra et al., 2017). Crystal structure of *Tg*NDK suggests that the protein forms a hexamer with conserved nucleotide binding sites. Pairwise structural alignment revealed that active site of *Tg*NDK may undergo similar conformational changes as its closest homolog, humanNM23-H2 transcription factor (Figure 3B). High structural homology to human transcription factor (NM23-H2) in the residues in the catalytic site indicate that selective inhibitors that do not act on human NM23-H2 will be needed. Possible strategies that will facilitate such selectivity include antisense, CRISPR, aptamer-based approaches where the DNA/RNA sequences are divergent, among others. When the dinucleotide is present in the active site, the residues in both *Tg* and Hs structures have similar side chain orientations (Figure 3B), suggesting inhibitor selectivity between the species might be difficult to achieve. On the other hand, when the dinucleotide is absent, the active site loop comprising of residues G59-F62 (*Tg* numbering) is shifted significantly (3–4 Å) between species. Additionally, *Tg*NDK residue K60 is residue R58 in the human ortholog, offering different hydrogen-bond capacities to putative ligands. These observations suggest opportunities for the selective design of *Tg*NDK inhibitors using structure-based molecular modeling techniques. Wang et al. reported selective NM23-H2 (human ortholog) inhibitors based on an isaindigotone scaffold (Wang et al., 2017). These compounds were subjected to molecular docking studies and were predicted to bind to the dinucleotide pocket. More so, they offer an obvious starting point for biological evaluation in *in vitro* parasite models and the subsequent design of selective *Tg*NDK chemical probes/ligands.

The next two enzymes, due to their sequential placement within the pentose-phosphate pathway, should be considered together. Ribulose phosphate 3-epimerase functions in the conversion of ribulose-5-phosphate into xylulose-5-phosphate, which is a reaction in the Calvin cycle. It is just downstream of the next target enzyme, ribose-5-phosphate isomerase, and is important for the development of a pool of NADPH, as well as in the pentose phosphate pathway that can convert monosaccharides like glucose into nucleotide precursor pentose sugars. This pathway has been of interest in targeting various organisms, including *P. falciparum* and *Trypanosoma cruzi* (Barrett, 1997; Bozdech and Ginsburg, 2005; Igoillo-Esteve et al., 2007). The biology of RPE has proven to be of particular interest in *T. cruzi* (Gonzalez et al., 2017). Indeed, the structure of RPE had already been characterized in *P. falciparum* (Caruthers et al., 2005). Determined crystal structure of *Tg*RPE revealed that Zn^2+^ binds in a putative active site. In addition, the presence of ${\text{SO}}_{4}^{2-}$ in the active site induces the β6–α5 loop to move from its presumed apo conformation in the absence of ligand, as seen in the human RPE homolog, to holo conformation in the ligand bound state (Figure 4B). Ribose-5-phosphate isomerase is the enzyme in the pentose phosphate pathway just upstream of ribulose phosphate 3-epimerase, and it has similar functions, though it catalyzes the transition from ribose-5-phosphate to ribulose-5-phosphate. It has been identified as a potential drug target in *P. falciparum*, one of the causative agents of malaria, due to its necessity in creating nucleotide precursors for DNA synthesis and for maintaining a large pool of NADPH for rapid replication and the metabolism necessary for the maintenance thereof (Holmes et al., 2006). It has also been of interest and targeted in *Mycobacterium tuberculosis* where inhibitors have been identified (Roos et al., 2005). Additionally, this pathway has been suggested as a drug target in trypanosomes, with identified compounds and multiple mechanisms of inhibition having been demonstrated, with current patents existing (de V. C. Sinatti et al., 2017). Perhaps the most interesting feature of the determined crystal structure of *Tg*RPI is the disordered region between residues 179–185 that comprises a part of what may appear to be an active site loop. These residues and 3_10_-helix η3 are unique to *Tg*RPI and might be important for catalysis (Figure 5A).

Ornithine aminotransferase, the final enzyme considered herein, is an enzyme involved in the urea cycle, TCA cycle, polyamine synthesis, and other pathways. It catalyzes a reversible reaction allowing interconversion of intermediates from ornithine to amino acids. Other enzymes which act in this pathway have been suggested as potential drug targets, and it is critical for maintaining proper amounts of free amino acids, so it was a good candidate for further study. Herein we found a modest phenotype on replication but raised antibody did not immunostain tachyzoites. Ornithine aminotransferase will be studied further in the future to resolve some of these questions and attempt to develop effective inhibitors, were it to prove to be essential. Structural analysis reveals that *Tg*OAT shares similar structural folds to known OAT enzymes (Figure 6). We have identified that *Tg*OAT shares a conserved cofactor and substrate-binding site with its closest homolog human GABA-AT. To gain insights into function of *Tg*OAT, we attempted to crystallize this enzyme with several different inhibitors and inactivators. As a result, three additional crystal structures of *Tg*OAT in complex with gabaculine (a potent inhibitor of human GABA-AT) and (S)-4-amino-5-fluoropentanoic acid have been determined (PDBs 5DJ9, 5E5I, and 5E3K). Therefore, we have performed a full kinetic and structural analysis. The details of the structure, substrate binding site, kinetic mechanism and function of *Tg*OAT will be described in our subsequent works.

Our approach described herein is a productive way to identify molecular targets and could potentially be useful for identifying small molecule inhibitors. *In silico* analysis of each enzyme's surface pockets/active sites suggest “druggable” areas for binding of putative small molecules. The recent discovery that CRISPR/Cas9 has future potential for treating HIV (Yin et al., 2017) also raises the possibility that expression of simply validated molecular targets can be eliminated by CRISPR/Cas9. SiRNA is another possible therapeutic modality, and is being studied in several infectious diseases, including Hepatitis C, Ebola, and viral encephalitis, among others (Kumar et al., 2006; Wan et al., 2014; Watanabe et al., 2014; Thi et al., 2015). As our studies suggest, vivoPMO inhibited *T. gondii* replication; antisense PMO is, therefore, another potential therapeutic modality that might effectively treat parasitic infection. The data presented in Figure 8 and CRISPR/Cas9 screen suggest a phenotype for *Tg*PGM, *Tg*RPE, and *Tg*RPI, but do not yet prove the targets are essential. With confirmation in the future, vivoPMO-based therapy with a less toxic molecular transporter has promise. The safety of PMO is well-documented in several clinical trials in treating genetic, cardiovascular, and infectious diseases including Duchenne muscular dystrophy (DMD), restenosis, and Marburg and Ebola hemorrhagic fevers (Kipshidze et al., 2007; Kinali et al., 2009; Cirak et al., 2011; Heald et al., 2014). It has even shown efficacy against a relative of *T. gondii*, the causative agent of malaria, *P. falciparum* (Augagneur et al., 2012). Eteplirsen (ExonDys51) is an FDA-approved PMO drug for treatment of DMD in patients who have confirmed mutation of the DMD gene that is amenable to exon 51 skipping. A clinical trial is underway using a cell-penetrating peptide conjugated eteplirsen (the PPMO technology) to increase intracellular delivery of eteplirsen for greater efficacy, lower dose and less frequent dosing (clincialtrials.gov: NCT03375255). With the advent of novel modalities, including antisense and small molecule inhibition, for the treatment of both active and latent infection, it may be possible to eradicate human *T. gondii* infection, and the successes achieved by physicians and scientists in combatting smallpox and dracunculiasis might extend to toxoplasmosis as well.

## Ethics statement

This study was carried out in accordance with the recommendations of The Home Office of the UK Government under the Animals [Scientific Procedures] Act 1986. All work was covered by License PPL60/4568, Treatment and Prevention of Toxoplasmosis with approval by the University of Strathclyde ethical review board.

## Author contributions

1JL, HN, and RM: Conceptualization. JL, HN, EF, AH, GM, YZ, ID, KF, LS, JR, KE, SD, CR, SW, MM, SM, CF, ES, RM, and WA: Data curation. JL, EF, AH, GM, YZ, and RM: Formal analysis. CR, RM, and WA: Funding acquisition. JL, HN, EF, AH, GM, YZ, ID, KF, LS, JR, KE, SD, CR, SW, MM, SM, CF, ES, RM, and WA: Investigation. JL, HN, EF, AH, GM, YZ, ID, KF, LS, JR, KE, SD, CR, SW, JM, HM, MM, SM, CF, ES, DS, DR, RM, and WA: Methodology. RM and WA: Project administration. CR, RM, and WA: Resources. RM and WA: Supervision. JL, EF, AH, GM, YZ, RM, and WA: Validation. JL, EF, AH, GM, YZ, and KE: Visualization. JL, EF, AH, GM, and RM: Writing-original draft. JL, HN, EF, AH, GM, YZ, ID, KF, LS, JR, KE, SD, CR, SW, JM, HM, MM, SM, CF, ES, DS, DR, RM, and WA: Writing-review and editing.

### Conflict of interest statement

RM, KE, HM, and JM are inventors on a U.S. patent (US8575126B2) that is focused on delivery of morpholino oligomers as a treatment for *Toxoplasma gondii infection*. JM is employed by Gene Tools, LLC, the company that manufactured the vivoPMO for this study. However, he took no part in data collection or analysis. RM has completed an unrelated literature review for Sanofi-Pasteur. The remaining authors declare that the research was conducted in the absence of any commercial or financial relationships that could be construed as a potential conflict of interest.

## Supplementary material

Supplementary Table 1 Data from replicate experiments demonstrate the efficacy of target-specific morpholino on parasite replication *in vitro*. Ratios of target-specific to off-target morpholino for all targets reflect statistically significant perturbation of parasite replication.
